# Prevalence and Associated Factors of Low Birth Weight among Term Newborns in Dilla Town, Southern Ethiopia

**DOI:** 10.1155/2020/8394578

**Published:** 2020-07-27

**Authors:** Tsegaye Mehare, Yewbmirt Sharew

**Affiliations:** ^1^Depratment of Biomedical Science, College of Medicine and Health Sciences, Dilla University, Dilla, Ethiopia; ^2^Depratment of Midwifery, College of Health Sciences, Debre Markos University, Debre Markos, Ethiopia

## Abstract

**Introduction:**

Birth weight has emerged as the pointer of infant wellbeing and the fundamental focus of infant health policy. Low birth weight predisposes newborns to loads of health disarray like underweight, stunting, hypoglycemia, hypothermia, mental retardation, physical, and neurodevelopment problems which results in high rates of morbidity and mortality. This study aimed to assess the prevalence and associated factors of low birth weights in term newborns.

**Objective:**

This study designed to assess the prevalence and associated factors of low birth weight among term newborns in Dilla town, Southern Ethiopia.

**Materials and Methods:**

Institutional based cross-sectional study design was used on 472 term newborns. The study setting includes three health centers (Dilla, Wallemi, and Tumetecha) within Dilla town and Dilla University referral hospital from September 1, 2018, to January 30, 2019. The study subjects were mothers with term newborns delivered in the above institutions and those delivered at home and received care within the first 24 hours after delivery in the above health care settings.

**Results:**

A total of 161 (34.1%) of the term newborns were low birth weight. Divorced mothers, rural residents, maternal age <20 years old, unwanted and unintended pregnancy, no ANC follow-up, no dietary counsel, no foliate supplementation, and cigarette smoking have shown an association with low birth weight of neonates.

**Conclusions:**

Even though the method of sampling used in this study has its confines, the prevalence of low birth weight in this study was higher than the estimate in Ethiopia. Therefore, it is recommended that special attention should be given to pregnant mothers to get adequate rest, additional diet, and antenatal services available and accessible to all pregnant women.

## 1. Introduction

Low birth weight (LBW) is defined as having a birth weight of less than 2.5 kilograms irrespective of the gestational age of the neonate according to World Health Organization (WHO) [1]. The chance of the newborn to survive, experience healthy growth, and development was highly determined by birth weight [2, 3]. Birth weight has emerged as the pointer of infant wellbeing and the fundamental focus of infant health policy [4]. LBW predisposes newborns to many health disarrays like underweight, stunting, hypoglycemia, hypothermia, mental retardation, physical, and neurodevelopment problems which results in high rates of morbidity and mortality [5, 6].

Usually, a baby born with less than 2.5 kilograms needs extra hospital care, and there are a regular alarm and uncertainty over future health outcomes [7]. In some developed countries, the proportion of low birth weight ranges from 2-3% whereas in many developing countries the proportion of LBW range 25-30% [8, 9].

The prevalence of LBW in Ethiopia was 17.3% [2] which is in line with developing countries 16.5% [10]. The information on risk factors of LBW was documented at different times and places in Ethiopia. A study on low birth weight among term newborns in Wolieta Sodo health facility showed that the proportion of LBW was 8.1% [11], a study done in Jimma zone reported 22.5% LBW [12]. A study in Gondar administrative zone at 12 health institutions in 1988 reported that the average prevalence of low birth weight was 11.1% [12]. Another study carried out to determine the prevalence and risk factors for low birth weight in Adwa general hospital revealed that among 424 term newborns 42 (10%) were LBW [13]; Axum and Laelay Maichew districts reported a LBW rate of 9.9% and 6.3%, respectively [14].

A study was done in Olkalou district hospital, Kenya, on factors associated with low birth weight among neonates reported that the proportion of LBW was 12.3% [15]. Moreover, the report from the study on the prevalence of low birth weight and its association with maternal body weight status from 2010 to 2011 in Uganda was (10%) a bit lower compared to Ethiopia [16]. Nevertheless, the proportion of skilled childbirth attendance is increasing in Ethiopia; we lack adequate evidence on maternal factors which would potentially predict birth outcomes of the newborns in the Dilla area which is characterized by diverse culture and dietary practices. Gedeo zone is one of the most densely populated areas in Ethiopia [17]. Even though, the area is evergreen throughout the year most of the households often suffer from chronic food insecurity even at times of crop harvest [18, 19]. The evidence on child health and nutritional status predictors particularly attached to maternal health during pregnancy and childbirth was very limited. Thus, the main aim of this study was to determine the prevalence and associated factors of LBW among term newborns in Dilla town, Gedeo zone, South Ethiopia.

## 2. Materials and Methods

### 2.1. Study Setting

The study was conducted in Dilla town (in three health centers and one referral hospital) from September 1, 2018, to January 30, 2019. Dilla is the capital city of Gedeo zone, SNNPR, Ethiopia; Dilla is 359 km and 100 km away from Addis Ababa and Hawassa, respectively. The town has 8 woredas and bordered by Sidama in the north, Oromiya in the south, east, and west. It is also bounded by the rivers Legedara north and east, Michelle in south and Waleme in the west. The town has a Woinadega type of weather condition with a total of 954,120 populations and has one referral hospital, three health centers owned by the government, and other private clinics.

### 2.2. Study Design

An institution-based cross-sectional study was conducted.

### 2.3. Study Population

Term newborn mother pair who delivered in the three health centers, one referral hospital, and those delivered at home but received care in the above health institutions within 24 hours of delivery during the study period were considered.

### 2.4. Sample Size

A sample size of 472 was calculated with the following assumptions: 8.1% expected proportion of low birth outcome took from similar Ethiopian study [11], 95% confidence level at 5% margin of error, and 10% none response rate considered. Since the total population (*N*) is less than 10,000, we made a correction formula and multiply by 4 to maximize the sample size. Then, the sample was proportionally allocated to government health facilities in Dilla town based on the number of previous childbirth attendance. Finally, all eligible postpartum mothers to term newborn pairs were consecutively recruited until subsamples for the facilities and the total sample for the study were achieved.

### 2.5. Inclusion

Term newborn with mother who delivered in the three health centers, one referral hospital, and those delivered at home but received care in the above health institutions within 24 hours of delivery.

### 2.6. Exclusion

Twins and neonates delivered from mothers who had known chronic or pregnancy-induced pathologies were excluded from the study.

### 2.7. Data Collection Procedures

Data were collected by trained midwives using structured interviewer-administered questionnaires from mothers. The weights of newborns were measured using the digital Seca balance scale (Germany) to the nearest 1 g. The scale was adjusted to the zero levels before weighing each newborn. Measurement was taken immediately after birth for those delivered in the health institutions, however, for those delivered at home took within the first 24 hours. The investigator supervised the data collectors randomly while interviewing the mothers and weighing newborn babies.

### 2.8. Data Quality Management

To guarantee the validity of the information gathered, midwives who are well experienced in delivery care were recruited and training was given on techniques of data collection before the actual data collection. The questionnaire was pretested on subjects with the same socio-demographic characteristics in another similar health institution before being applied to the study population, and the necessary modifications were made before the main study. The investigators reviewed and checked the data each day after data collection for its completeness and accuracy.

### 2.9. Ethics Approval and Consent to Participate

Ethical clearance was obtained from the Institutional Review Committee of Dilla University, College of Medicine and Health Sciences. Official letter of cooperation will be distributed to each of the data collection sites. Before the questionnaire, the aims and objectives of the study were clearly explained to the participants. Verbal informed consent was obtained from each study participant before data collection. Confidentiality of the data was maintained by excluding their identifiers. All participants were involved in this study only with their willingness.

### 2.10. Statistical Analysis

The entire data collected using a structured questionnaire were validated, edited, coded, and entered into SPSS version 23. Followed by checking for any inconsistencies, descriptive analysis was performed. Descriptive statistics, such as frequencies, and percentages were used to describe the maternal socio-demographic and obstetric history, sex, and weight of the neonates. A chi-square test was used to find the potential association of dependent and independent variables. Bivariate logistic regression analysis was done to decide whether there is an association between low birth weight and different factors to select nominee variables for multivariate logistic regression. Variables with *p* values of up to 0.20 in the bivariate logistic regression analysis were identified and fitted to the multiple logistic regression analysis to identify the independent effects of each variable to the outcome variable. The odds ratio with 95% confidence intervals (CI) was calculated to distinguish the occurrence and strength of associations, and statistical significance was affirmed if *p* < 0.05.

## 3. Results

Socio-demographic characteristics: in this study, a total of 472 singleton deliveries from the three health centers and one referral hospital were included giving a response rate of 100%. The socio-demographic characteristics of mothers studied revealed that 143 (30.3%) were 20-30 years, 120 (25.4%) were between 31 and 40 years of age, 108 (22.9%) were less than 20 years of age, while the remaining 101 (21.4%) were above 40 years of age. The predominant religion of the study population was found to be Protestant 244 (51.7), followed by Orthodox 98 (20.8%), and Muslim 75 (15.9%), while the rest were neither Christians nor Muslims. The predominant ethnic group was 288 (61%) Gedeo followed by Gurage 73 (15.5%). Analysis of educational levels of mothers showed that the majority 257 (54.4%) had elementary education, 111 (23.5%) had no formal education, while the rest 104 (22%) had secondary school and above levels of educational. Concerning monthly family income among the study population, 242 (51.3%) earned less than 2001 ETB, followed by 119 (25.2%) who earned between 2001 and 4000 ETB, while the rest 111 (23.5%) earned greater than 4000 ETB. The mothers' response to their occupational status revealed that 107 (22.7%) were farmers, 105 (22.2%) were government employees, 99 (21%) were merchants, 84 (17.8%) were daily workers, while the remaining 77 (16.3%) were housewives. Among the mothers studied, majority 221 (46.8%) were married, 130 (27.5%) were unmarried, and the rest 121 (25.6%) were divorced. The majority of the respondents, 369 (78.2%), were urban, and 103 (21.8%) were rural residents (Table 1).

### 3.1. Maternal Obstetric and Health Service Utilization

Two hundred twenty-three (47.2%) of mothers were primiparous, 128 (27.1%) of mothers gave 2–4 births, and the rest gave 5 and more births. Concerning pregnancy type among the study population, 173 (36.7%) were wanted but unintended, followed by 168 (35.6%) who were unwanted and unintended, and the rest 131 (27.8%) were wanted and intended. The majority, 235 (49.8%), of the mothers gave birth to the current newborn with less than two years (<24 months) after previous childbirth. The majority, 225 (47.7%), had no antenatal care (ANC) follow-up, 124 (26.3%) had complete follow-up, and the rest of 123 (26.1%) had 1-3 ANC follow-up during recent pregnancy. 307 (65%) mothers drank alcohol during pregnancy, and the rest 165 (35%) had not drunk alcohol during pregnancy. 337 (71.4%) mothers had no cigarette smoking habits during recent pregnancy, whereas 135 (28.6%) had a cigarette smoking experience. 369 (78.2%) mothers affirmed for getting dietary advice during ANC follow-up. The majority of the respondents, 345 (73.1%), took iron-folic acid. Analysis of sex of neonates showed that slightly more than half, 240 (50.8%), were female, while the rest 232 (49.2%) were male (Table 2).

### 3.2. Prevalence and Associated Factors of Low Birth Weight (LBW)

In this study, 34.1% of term neonates were found to be LBW. The bivariate logistic regression analysis showed that maternal age, residency, educational status, occupation, marital status, birth interval, pregnancy type, ANC follow-up, dietary counsel, foliate supplementation, alcohol drinking, and cigarette smoking were associated with a low birth weight of neonates. After adjustments for possible effects of confounding variables, ANC follow-up, residency, dietary counsel, foliate supplementation, cigarette smoking, pregnancy type, marital status, and maternal age were significantly and assertively associated with low birth weight.

No ANC follow-up during pregnancy showed a significant association with low birth weight. In epidemiological explanation, this showed us that mothers who had no ANC follow-up were 6.83 times more likely to give low birth weight term neonates as compared to those mothers who had complete ANC follow-up during pregnancy with (AOR: 6.83 95% CI 3.57, 13.05). Being a rural resident was strongly associated with low birth weight. Those mothers who were rural residents were 6.78 times more likely to give term low birth weight neonates as compared to their counterparts with (AOR: 6.78 95% CI 3.51, 13.08). The likelihood of delivering low birth weight neonates among mothers who did not get dietary counsel was 6.21 times (AOR: 6.21 95% CI 3.21, 12.02) more compared with mothers who got dietary counsel. Those mothers who did not take folate supplementation during pregnancy were 5.48 times (AOR: 5.48 95% CI 2.93, 10.25) as likely to give term low birth weight neonate as those mothers who took foliate. Furthermore, cigarette smoking during pregnancy was a significant factor associated with low birth weight neonates. Mothers who smoke a cigarette during pregnancy were 4.35 times more likely to give term low birth weight neonate as compared with those mothers who did not smoke with (AOR: 4.35 95% CI 2.46, 7.69). Unwanted and unintended pregnancy was strongly associated with low birth weight. Those mothers who got pregnant unwanted and unintendedly were 3 times more likely to give term low birth weight neonates compared to mothers who got pregnant in a wanted and intendedly with (AOR: 3.03 95% CI 1.64, 5.58). Being divorced and age <20 years old mothers were another significant factor associated with low birth weight. Divorced as well as age <20 years old mothers were 3.12 and 4 times more likely to give term low birth weight neonates as compared to married and age ≥40 years old mothers, respectively, for being divorced (AOR: 2.57 95% CI 1.43,4.61) and age <20 years old (AOR: 2.46 95% CI 1.21, 5.00) (Table 3).

## 4. Discussion

The prevalence of LBW in this study was 34.1%, relatively higher than the estimate of LBW from the Ethiopian Demographic Health Survey (EDHS) 2011 (28%) [20] and Endalamaw et al. [2]. It was also higher than studies conducted on birth weight in Wolaita Sodo and Adwa general hospital in which the prevalence of LBW was found to be 8.1% in Woliata Sodo [11] and 10% in Adwa general hospital [13]. Another study undertaken in Jimma Zone on 645 births reported the prevalence rate of LBW (22.5%) [12]. In 2018, the pooled prevalence of LBW in Ethiopia was reported to be 17.3% [2], while a study was undertaken to find out the prevalence and factors associated with low birth weight delivery in Axum and Laelay Maichew districts revealed that among the 520 live births 9.9% were LBW babies [14]. The possible rationalization of high prevalence in the current research could be the internal displacements due to ethnic clash occurred in the border between the Gedeo zone and the Guji zone for the last two years. The other possible justification for differences might be due to poor health education provided by health extension workers to the study participants in the current study area. Also, the current result is higher compared with LBW prevalence (22.30%) reported from Tanzania during the year 1976-1977 [21].

In this study, the risk of delivering LBW term newborn was found to be significantly higher in those mothers who were residing in rural areas than those residing in urban areas. This may be related to the lifestyles of rural dwellers such as poor balanced diet habits and drinking local alcohol, which is commonly practiced by young women of rural areas. Another possible explanation for the difference might be the study settings, such as access to the health facility and acquiring maternal care education through media is more common in urban than rural. Maternal age was an important determinant of the low birth weight of term neonates in this study. Mothers who had less than 20 years at the time of delivery gave a higher proportion of LBW babies as compared to mothers whose age were greater than or equal to 40 years old. This result is similar to the study conducted in Adwa [13], systematic review, and meta-analysis in Ethiopia [2], Nepal [22], and India [23]. In this study, marital status enlightened a significant association with low birth weight. Divorced mothers had higher odds to delivered term LBW neonates than mothers who were married.

The pregnancy type was also another associated factor for the term LBW. Those mothers whose pregnancies were unwanted and unintended gave more low birth weight neonate as compared to those mothers whose pregnancies were wanted as well as intended. This finding is in line with studies carried out on low birth weight and associated factors in Axum and Laelay Maichew districts [14], Adwa general hospital [13]. This might be explained by the fact that mothers might not be ready psychologically, economically, and nutritionally; therefore, they did not take extra diet during the entire pregnancy period. Similarly, those mothers who did not take folate supplementation during pregnancy were more likely to give term LBW neonates as compared to those mothers who took foliate. The protective function of foliate supplementation to the low birth weight of neonate is supported by the previous study conducted in Adwa general hospital [13], Nepal [22], and America [24]. The physiological mechanism of iron supplementation on birth weight is not understood; however, there are two hypotheses about improvements in birth weight due to iron supplements [24]. First, iron supplementation helps to improve appetite which improves the overall nutritional status of mothers. Second, iron deficiency anemia leads to change in norepinephrine, cortisol, and corticotrophin that result in oxidative stress to fetal growth which is reduced by iron supplementation [24, 25].

Furthermore, in this study, we observed that those mothers who had not ANC follow-up and dietary counsel were more likely to give term LBW neonates as compared to mothers who had complete ANC follow-up and dietary counsel, respectively. The current findings are supported by findings from 2006 to 2011 NDHS [22], Axum and Laelay Maichew districts [14], Jimma [12], and Gondar [26]. The possible explanation could be ANC visits are likely to influence improvements in dietary practices, monitor and encourage recommended weight gain during pregnancy, and improve neonatal outcomes.

Lastly, smoking was another maternal factor significantly associated with the term LBW. Mothers who had a habit of smoking during pregnancy were more likely to give LBW neonates as compared with nonsmoker mothers. There was a dissimilar result done by Cogswell et al. [24].

## 5. Conclusion and Recommendation

The magnitude of low birth weight in Dilla town was too high. ANC follow-up, residency, dietary counsel, foliate supplementation, cigarette smoking, pregnancy type, marital status, and maternal age showed significant association with LBW neonates. Thus, it is recommended that special attention should be given to pregnant mothers to get adequate rest and additional diet, and making antenatal services available and accessible timely to all pregnant women is recommended to minimize the prevalence of low birth weight.

## Figures and Tables

**Table 1:** Sociodemographic characteristics of the mothers.

Characteristics	Frequency	Percent
Age category in (years)		
<20 yrs	108	22.9
20-30 yrs	143	30.3
31-40 yrs	120	25.4
>40 yrs	101	21.4
Residency		
Urban	369	78.2
Rural	103	21.8
Marital status		
Married	221	46.8
Divorced	121	25.6
Unmarried	130	27.6
Level of education		
No formal education	111	23.5
Primary education	147	31.2
Grade 7-8 education	110	23.3
Secondary and above education	104	22.0
Occupation		
Employ	105	22.2
Merchant	99	21.0
Farmer	107	22.7
Housewife	77	16.3
Daily worker	84	17.8
Monthly income (in birr)		
<1000 ETB	101	21.4
1000-2000 ETB	141	29.9
2001-4000 ETB	119	25.2
>4001ETB	111	23.5
Ethnicity		
Gedeo	288	61.0
Amhara	29	6.1
Oromo	72	15.3
Gurage	73	15.5
Others	10	2.1
Religion		
Protestant	244	51.7
Orthodox	98	20.8
Muslim	75	15.9
Others	55	11.7

**Table 2:** Obstetric, nutrition-related characteristics of mothers and sex of neonates.

Characteristics	Frequency	Percent
Parity		
Po	223	47.2
2-4 births	128	27.1
≥5 births	121	25.6
Birth interval		
<2 yrs	235	49.8
2-3 yrs	109	23.1
≥4 yrs	128	27.1
Sex of neonate		
Female	240	50.8
Male	232	49.2
Type of pregnancy		
Unwanted and unintended	168	35.6
Wanted but unintended	173	36.7
Wanted and intended	131	27.8
ANC follow-up		
No follow-up	225	47.7
1-3 follow-up	123	26.1
≥4 follow-up	124	26.3
Dietary counsel		
Yes	369	78.2
No	103	21.8
Foliate supplementation		
Yes	345	73.1
No	127	26.9
Alcohol drink at pregnancy		
No	307	65.0
Yes	165	35.0
Smoking status		
No	337	71.4
Yes	135	28.6

**Table 3:** Bivariate and multivariate logistic regression analysis of factors associated with low birth weight neonates, Dilla town, Southern Ethiopia.

Variables	LBW		COR (95% CI)	AOR (95% CI)
	Yes	No		
Maternal age				
<20 yrs	56 (51.9%)	52 (48.1%)	2.81 (1.58,4.99)	2.46 (1.21,500)∗
20-30 yrs	42 (29.4%)	101 (70.6%)	1.08 (0.61,1.91)	1.1 (0.56,2.17)
31-40 yrs	35 (29.2%)	85 (70.8%)	1.07 (0.59,1.93)	0.88 (0.43,1.78)
≥41 yrs	28 ((27.7%)	73 (72.3%)	Ref	Ref
Level of education				
No formal education	58 (52.3%)	53 (47.7%)	2.83 (1.60,4.99)	2.21 (0.09,52.07)
Primary education	42 (28.6%)	105 (71.4%)	1.03 (0.59,1.81)	0.93 (0.04,24.82)
Grade 7-8 education	32 (29.1%)	78 (70.9%)	1.06 (0.59,1.92	2.39 (0.06,90.08)
≥secondary education	29 (27.9%)	75 (72.1%)	Ref	Ref
Residency				
Urban	114 (30.9%)	255 (69.1%)	Ref	Ref
Rural	47 (45.6%)	56 (54.4%)	1.88 (1.20,2.93)	6.78 (3.51,13.08)∗∗∗
Occupation				
Employ	39 (37.1%)	66 (62.9%)	Ref	Ref
Merchant	27 (27.3%)	72 (72.7%)	0.64 (0.35,1.15)	0.68 (0.32,1.44)
Farmer	29 (27.1%)	78 (72.9%)	0.63 (0.35,1.13)	0.59 (0.28,1.25)
Housewife	27 (35.1%)	50 (64.9%)	0.91 (0.49,1.69)	0.73 (0.33,1.64)
Daily worker	39 (46.4%)	45 (53.6%)	1.47 (0.82,2.63)	1.43 (0.66,3.11)
Marital status				
Divorced	88 (39.8%)	133 (60.2%)	1.87 (1.16,3.00)	2.57 (1.43,4.61)∗∗
Never married	39 (32.2%)	82 (67.8%)	1.34 (0.78,2.34)	1.56 (0.80,3.06)
Married	34 (26.2%)	96 (73.8%)	Ref	Ref
Pregnancy type				
Unwanted and unintended	77 (45.8%)	91 (54.2%)	2.51 (1.53,4.13)	3.03 (1.64,5.58)∗∗∗
Wanted but unintended	51 (29.5%)	122 (70.5%)	1.24 (0.74, 2.07)	1.26 (0.68,2.34)
Wanted and intended	33 (25.2%)	98 (74.8%)	Ref	Ref
Birth interval				
<2 yrs	119 (50.6%)	116 (49.4%)	5.89 (3.39,10.20)	3.05 (0.0.59,15.8)
2-3 yrs	23 (21.1%)	86 (78.9%)	1.53 (0.79,2.99)	4.03 (0.80,20.37)
≥4 yrs	19 (14.8%)	109 (85.2%)	Ref	Ref
ANC follow up				
No follow-up	117 (52%)	108 (48%)	5.31 (3.11,9.09)	6.83 (3.57,13.05)∗∗∗
1-3 follow-up	23 (18.7%)	100 (81.3%)	1.13 (0.59,2.17)	1.04 (0.49,2.20)
≥4 follow up	21 (16.9%)	103 (83.1%)	Ref	Ref
Dietary counsel				
Yes	113 (30.6%)	256 (69.4%)	Ref	Ref
No	48 (46.6%)	55 (53.4%)	1.98 (1.27,3.09)	6.21 (3.21,12.02)∗∗∗
Foliate supplementation				
Yes	100 (29%)	245 (71%)	Ref	Ref
No	61 (48%)	66 (52%)	2.26 (1.49,3.44)	5.48 (2.93,10.25)∗∗∗
Alcohol drink				
No	86 (28%)	221 (72%)	Ref	Ref
Yes	75 (45.5%)	90 (54.5%)	2.14 (1.44,3.18)	1.91 (0.78,4.66)
Cigarette smoking				
No	99 (29.4%)	238 (70.6%)	Ref	Ref
Yes	62 (45.9%)	73 (54.1%)	2.04 (1.35,3.08)	4.35 (2.46,7.69)∗∗∗

∗*p* < 0.05, ∗∗*p* < 0.01, ∗∗∗*p* < 0.001.

## Data Availability

The datasets analyzed during the current study are available from the corresponding author on reasonable request.

## Abbreviations

LBW: Low birth weight
AOR: Adjusted odds ratio
CI: Confidence interval
SPSS: Statistical package for social science
ANC: Antenatal care
SNNPR: South Nation Nationalities and People's Region
WHO: World Health Organization.
